# Evidence of Biomass Smoke Exposure as a Causative Factor for the Development of COPD

**DOI:** 10.3390/toxics5040036

**Published:** 2017-12-01

**Authors:** Sarah J. Capistrano, David van Reyk, Hui Chen, Brian G. Oliver

**Affiliations:** 1 School of Life Sciences, University of Technology Sydney, Ultimo, NSW 2007, Australia; Sarah.Capistrano@student.uts.edu.au (S.J.C.); David.VanReyk@uts.edu.au (D.v.R.); Hui.Chen-1@uts.edu.au (H.C.); 2 Respiratory Cellular and Molecular Biology, Woolcock Institute of Medical Research, The University of Sydney, NSW 2037, Australia; 3 Emphysema Center, Woolcock Institute of Medical Research, The University of Sydney, NSW 2037, Australia

**Keywords:** biomass, emphysema, COPD

## Abstract

Chronic obstructive pulmonary disease (COPD) is a progressive disease of the lungs characterised by chronic inflammation, obstruction of airways, and destruction of the parenchyma (emphysema). These changes gradually impair lung function and prevent normal breathing. In 2002, COPD was the fifth leading cause of death, and is estimated by the World Health Organisation (WHO) to become the third by 2020. Cigarette smokers are thought to be the most at risk of developing COPD. However, recent studies have shown that people with life-long exposure to biomass smoke are also at high risk of developing COPD. Most common in developing countries, biomass fuels such as wood and coal are used for cooking and heating indoors on a daily basis. Women and children have the highest amounts of exposures and are therefore more likely to develop the disease. Despite epidemiological studies providing evidence of the causative relationship between biomass smoke and COPD, there are still limited mechanistic studies on how biomass smoke causes, and contributes to the progression of COPD. This review will focus upon why biomass fuels are used, and their relationship to COPD. It will also suggest methodological approaches to model biomass exposure in vitro and in vivo.

## 1. Overview

**Figure 1:**
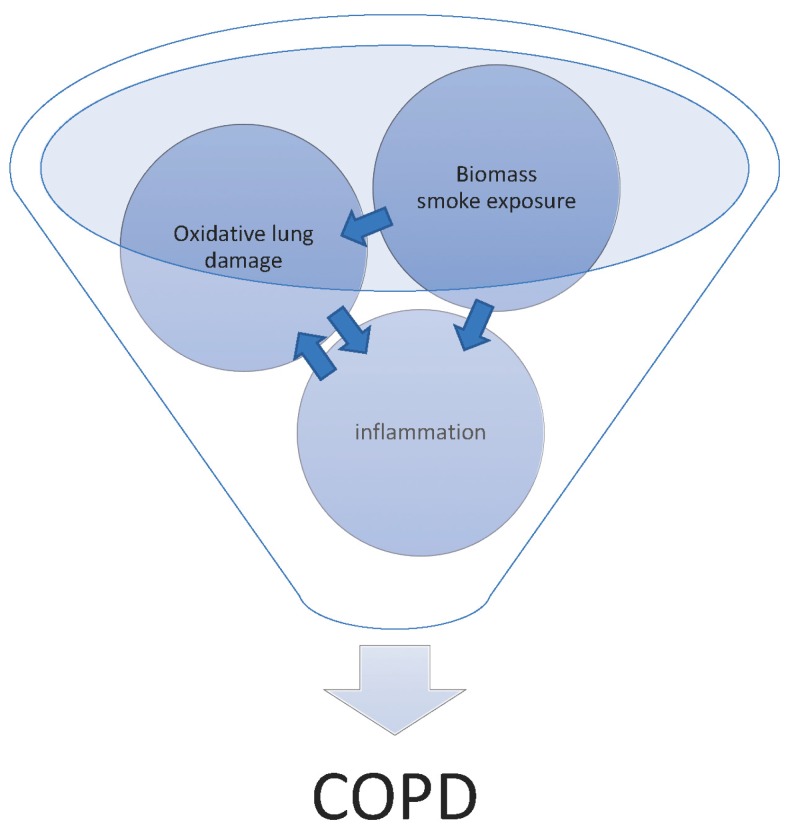
Graphical representation of the interaction of biomass smoke, oxidative lung damage and inflammation in the initiation of chronic obstructive pulmonary disease (COPD).

Chronic obstructive pulmonary disease (COPD) can be thought of as the physical manifestation of the pulmonary response to chronic inhalation of noxious particles. The prevailing theory is that these noxious particles induce an inflammatory response and tissue damage, and in susceptible individuals, these result in COPD. Susceptibility to COPD may depend upon epigenetic reprogramming of lung cells [1], inheritable genetic susceptibility [2], and intrinsic differences in lung structure [3], with the relative contribution of each being unknown. Airway inflammation is a key immediate immunological response after exposure, which is a key marker for the pathological effects of this disease. This involves the recruitment of inflammatory cells such as neutrophils, macrophages, eosinophils and T cells into the airways, which all contribute to tissue damage and airway remodeling. In comparison to healthy people, COPD patients have an exaggerated inflammatory response; for the same amount of stimulus, there are more inflammatory cells and higher pro-inflammatory cytokine levels in the lungs [4]. Clinically the severity of COPD is classified by the amount of airflow obstruction. Pathologically COPD is characterised by three interrelated processes: remodeling of the small airway walls, loss of small airways, and emphysema—the destruction of alveolar structure leading to airspace enlargement and loss of elastic recoil and ultimately of peribronchiolar attachments. Hogg et al. carried out a comprehensive pathological assessment of small airway thickening in COPD in which they inflammatory cell increased according to the severity of COPD [5]. Inflammatory cells release high levels of reactive oxygen species (ROS) in COPD, which is also found in biomass smoke, and induce oxidative stress (see Figure 1). This process can then activate proteases such as matrix metalloproteinases and neutrophil elastase, and increase inflammatory cell influx at the same time [6,7]. In turn, these cells will further release more proteolytic enzymes, which can be activated and cause break down of connective tissues in the lung [8,9]. Therefore, airway inflammation, oxidative stress and protease/antiprotease imbalance are interlinked and all contribute to the development of COPD. 

Lung pathology involves the destruction of the parenchyma (i.e., emphysema), mucus hypersecretion and thickening, fibrosis, occlusion and loss of the small airways. The relative extent of each of these varies from person to person. These pathological changes are manifested as symptoms associated with airflow limitation such as coughing, wheezing, and shortness of breath [10]. Disease heterogeneity [11,12,13], both in terms of lung pathology, the immunological response, and symptoms leads to simplification in clinical trial design and in vitro and in vivo experimentation. For example, because of the strong association with the risk of COPD, most investigators have focused upon cigarette smoke exposure to understand the pathophysiology of COPD. Biomass smoke exposure has been posited as the greatest risk factor for the development of COPD globally [14], but despite this, there have been remarkably few mechanistic studies on how biomass smoke causes, and contributes to the progression of COPD. 

## 2. Prevalence of Biomass Smoke Exposure

Biomass smoke is one of the major air pollutants and contributors of household air pollution worldwide. It is considered one of the leading environmental risk factors of several diseases, including COPD and acute lower respiratory disease, and is thought to cause 4 million deaths annually across the globe [15,16]. Biomass smoke is the result of the combustion of different types of fuels such as wood, animal dung, and crop residues undertaken to create the energy necessary for cooking and heating in many households worldwide [17]. Recent estimates are that 3 billion people rely on biomass fuels for domestic purposes [18]. The proportion of households using biomass fuels varies substantially across the globe (and even in the same continent) due to biomass availability and relative costs compared with other energy sources such as electricity and liquid petroleum gas. This makes quoting percentages per region less informative although there is a clear trend for greater use in the undeveloped and developing world [19]. 

There are many reasons why biomass fuels are used. In developing countries where poverty is prevalent, burning biomass fuels is a cheaper alternative compared to liquefied petroleum gas or electricity. Also, biomass fuels are also more accessible, especially for people living in rural places [20,21]. Current predictions are that domestic consumption of biomass fuels will remain substantial for decades to come, particularly in rural areas [20,21]. The most common type of biomass used worldwide is wood; however, most people in developing countries still use a combination of different solid fuel sources depending on availability [17,22]. In developing countries women and children have the highest biomass smoke exposure due to cultural practices such as indoor cooking in housing with very poor air ventilation. The absence of chimneys or pipes prevents the smoke venting outside and therefore, particles become trapped and diffuse into the surroundings [23,24]. During the burning of these fuels, people indoors can be exposed with up to 30,000 μg/m^3^ of particulate matter (PM) sized 10 μm or smaller (i.e., PM10 which includes PM2.5 μm or smaller (PM_2.5_), while an average concentration throughout the day is approximately 300–5000 μg/m3 [25]. We have previously evaluated PM production from biomass and tobacco cigarettes and under laboratory conditions have found that the profile of PM production is very similar [26]. The concentration of PM from second-hand smoking can be up to 704 ug/m^3^ [27].

However, one stark difference to exposure to second-hand tobacco smoke is that as a result of remaining indoors, woman and children are exposed for about 3 to 7 h a day to biomass smoke [28]. The WHO guideline [29] for PM10 concentration exposure is only 50 μg/m^3^ for a 24 h period, which is extremely low compared to observed concentrations indoors where biomass fuels are burnt. The Global Burden of Disease 2010 study found that household air pollution is the second highest risk factor of ill health for women and girls globally [30]. 

Exposure to biomass smoke is not exclusively an issue in the developing world. Indoor and outdoor air pollution in developed countries was previously estimated to contribute to 23% of the total global exposure to particulate matter pollution [21]. Use of indoor wood fires, seen as a cheaper, renewable (and possibly more “natural”) alternative to electrical and gas heating; is increasing in developed countries thus increasing biomass smoke exposure [31]. In 2011, in Australia, 5% of households surveyed used wood stoves for indoor heating. The use of wood fire as a source of heating between different states did not vary dramatically with the exception of Tasmania where wood fire heating was used in 20% of households [32]. Domestic use of wood fires in developed countries shares some features with that of developing countries including that its use is more common amongst the rural poor. 

Interestingly, biomass fuel use in developed countries has been shown to increase during times of recession [33]. However, in the developed countries, use of biomass fuel is more seasonal since the principal function is indoor heating; there is better venting of the resultant smoke and duration of exposure to children is usually less because during the day they are attending schools [34]. With regard to cooking, in developed countries, biomass fuels are may be chosen for the flavor that they impart during cooking processes (e.g., barbecues, smoked meats, wood-fired pizza). Outside the home, some occupations such as firefighters involve high exposure rates to biomass smoke [35]. While in countries with substantial park and bushlands, such as Australia, Canada and the USA, an additional, seasonal source of biomass smoke are bushfires. Across the globe intentional use of fire as part of agricultural practices is another seasonal outdoor contributor to biomass smoke [31].

## 3. Biomass Smoke as a Toxic Air Pollutant

Biomass smoke has been shown to consist of over 200 different compounds, which includes a significant number of toxic compounds. Some of these include carbon monoxide (CO), varying sizes of particulate matter (PM), mostly PM10; sulphur and nitrogen oxides, polycyclic aromatic hydrocarbons (PAH), aldehydes, free radicals and non-radical oxidising species; and volatile organic compounds [20,23,31]. Many of these compounds cause respiratory diseases while some are carcinogenic (reviewed in [36]). The exact chemical composition of biomass smoke is dependent upon the fuel type, the temperature of burning, whether an open fire or incinerator is used, and local conditions (e.g., wind speed, humidity, indoor or outdoor fires). It is the author’s opinion that whilst studying the toxicological and health effects of individual components of biomass smoke is informative, people are exposed to a toxic mixture of all components and it is difficult to extrapolate individual effects to such complex mixtures. As an example, Table 1 gives the top 25 chemical groups in terms of g/kg of wood smoke. In comparison, tobacco smoke constituents have been intensively studied, and whist much more is known about the contents of the smoke, it is not known which of the 70 carcinogens is more likely to cause lung cancer in any given individual. 

In epidemiological studies if source sampling is not available it is possible to estimate biomass exposure levels using tracer chemicals such as levoglucosan. Levoglucosan is a cellulose pyrolysis product, and has been used as a general organic tracer for wood smoke particles [37,38]. Such tracer chemicals allow exposures and risk of diseases to be calculated even when people are living several kilometers away from the sauce of the biomass smoke.

## 4. Biomass Smoke-Induced COPD

Biomass smoke exposure is a prominent risk factor for developing several airway diseases. For example relative to non-exposed people, those exposure to biomass smoke have an odds ratio of 2.44 (95% CI, 1.9–3.33) for developing COPD [40]. While among women over 30 who were predominantly undertaking domestic duties in rural areas the relative risk for COPD was estimated as either 3.2 (95% CI 2.3–4.8) [41] or 2.14 (95% CI 1.78–2.58) [42]. In children under five years from developing countries, the relative risk for acute lower respiratory disease was estimated at 2.3 (95% CI 1.9–2.7 [41]) or 1.78 (95% CI 1.45–2.18) [43]. While for lung cancer in women over 30 exposed to coal smoke the relative risk was 1.9 (95% CI 1.1–3.5) [41]. There is also evidence for adverse impacts in terms of low birth weight, cardiovascular disease and early mortality [17,22,31,44]. 

Pathologically biomass induced COPD is distinct from cigarette smoke induced COPD. Rivera et al. were the first to carry out an elegant study into the pathology of biomass induced COPD [45]. In comparison to cigarette smoke-exposed women with COPD, the lungs of biomass smoke exposed women with COPD had more pigment deposition and fibrosis (collectively referred to as bronchial anthracofibrosis), and thicker pulmonary arterial intima, but had reduced emphysema. Bronchial anthracofibrosis is not unique to biomass smoke induced COPD [46], and for example occurs in around 50% of people with tuberculosis [47]

## 5. Life-Long Exposure to Biomass Smoke in COPD Patients

Adults with COPD linked to biomass exposure in developing countries would typically have a life-long exposure to biomass smoke, from when they were children until death, and even after a COPD diagnosis, especially in older women having a higher risk because of their increased cumulative exposure. Thus, the typical person suffering from COPD linked to biomass exposure is an elderly woman who most likely grew up in a rural area in an underdeveloped or developing country. Despite this, there are still limited studies on patients with COPD within these areas where biomass PM concentrations are abnormally high compared to most developed countries. In terms of public health measures, intervention studies such as the implementation of low-cost, improved wood-burning stoves (which reduce personal exposures of PM and CO levels), and provision of liquefied petroleum gas stoves, have been carried out. Compared to open fires, the use of efficient wood stoves was shown to reduce up to 71% of particulate matter of sizes of 2.5 μm or smaller (PM_2.5_) concentrations near the stove area [48]. Another study showed significantly lower risk of respiratory symptoms and reduced decline in forced expiratory volume (FEV1) after a year of using efficient wood stoves called “Patsari stoves” in Mexico [49]. Due to the persistent exposure of biomass smoke despite developing COPD in patients, it is important to investigate its effects on the disease progression.

## 6. How Does Biomass Smoke Exposure Contribute to the Development of COPD?

Regarding COPD, the global impact of biomass smoke exposure strongly supports further research into the mechanisms by which this significant household pollutant could induce COPD in susceptible individuals. Researchers have utilised cellular-, animal- and human exposure models to investigate these mechanisms.

### 6.1. In Vitro Studies

Studies that rely upon the exposure of either whole biomass smoke (or individual and/or combinations of toxic components) to cells in vitro allow for a more precise elucidation of the pathological mechanisms involved. Where such in vitro models of biomass smoke exposure are evaluated to sufficiently reflect central features of the disease in question, then such models provide the first stage to evaluate both the efficacy of potential therapeutic agents and identify genetic components of disease susceptibility. Comparisons of the responses elicited between cells isolated from healthy donors and those with either disease (or increased risk of the disease) provide an ethically acceptable alternative to in vivo exposure studies. However, there are several issues, which limit the impact of such in vitro studies. One is a technical issue with regard to the delivery of the smoke. It is standard practice with in vitro studies for the cells to be fully submerged in liquid growth media often containing serum. Infusing culture medium with biomass smoke will potentially lead to the consumption of active components of biomass smoke and the generation of potentially more toxic products from reactions between biomass smoke components and components of the culture medium. This will be of particular concern for studies looking at the responses of alveolar and airway epithelial cells. A second caveat in the case of studies of the COPD being that the disease is the end result of chronic exposure to noxious agents. Both the nature of the biomass smoke and the inclusion of a known noxious gas (i.e., cigarette smoke) need to be considered as well as the duration of stimulation. Of interest is the recent study from Happo et al. where they found that in vitro responses of a macrophage cell line to PM exposure (from wood combustion) varied with the time of exposure from 2 to 32 h [50]; whereas humans can be exposed to biomass PMs delivery continuously in an indoor environment, which also accumulate in the airway. 

While keeping the limitations in mind, it remains that there have been significant findings made regarding the impact of biomass smoke using in vitro systems. Biomass smoke exposure has been shown to be pro-inflammatory. This response is observed with both smoke from the combustion of wood [40,41] and animal dung [42]. Since there are many common components in these smokes, it may suggest that a common component(s) of the smoke initiates inflammation [51,52]. This notion is supported by a study where consistent inflammatory responses were found to 6 different types of dung [43]. Biomass smoke-induced COPD may not simply be a by-product of biomass-smoke induced inflammation as in vitro exposure of epithelial cells to biomass PM from wood epigenetically modified the transcriptome resulting in altered gene expression [45]. It is also important to consider the impact of biomass-smoke within the milieu of pro-inflammatory constituents that constitute household pollutants [33]. In addition, components from biomass smoke may work synergistically with other pro-inflammatory agents as recently Capistrano et al. have demonstrated that biomass smoke exposure of human pulmonary fibroblasts in vitro resulted increased production of extracellular matrix proteins that, in synergy with exposure to rhinovirus, resulted in a more inflammatory phenotype [44]. These findings are of signficance given the airway remodelling that is a common feature of COPD [53].

### 6.2. Animal Studies

Animal models of biomass exposure are particularly usefully to examine the response to biomass smoke in systems that are more complex. Particularly the interplay of immune and respiratory systems given previous in vitro studies demonstrating the modulation of leukocyte function in response to biomass smoke exposure [50,54,55] and the role of leukocytes in the pathogenesis of inflammatory diseases like COPD. They also allow for assessing more chronic exposure; while selective breeding and the availability of gene knockout strains allow the development of models of inheritable risks. Models have varied regarding the species used (for example rabbits [56], mice [57,58,59], rats [60,61,62], guinea pigs [63,64] as well as larger species such as dogs and sheep [65,66]), the nature of the exposure system (smoke from biomass, exposure to PM), and the length of the exposure (acute or chronic). In reporting on the findings of The Toxicology and Animal Study Design Workgroup at the 2009 International Biomass Smoke Health Effects (IBSHE) conference, Migliaccio and Mauderly stated that after reviewing many studies, it was only studies modeling COPD, emphysema, and the potential CNS effects that the working group were not confident about with regard to providing reliable data demonstrating the adverse effects of biomass smoke exposure [67]. However, from the perspective of pulmonary disease researchers it is clear that while outcome measurements from numerous animal studies have varied, such studies remain valid as they generally explore inflammatory mechanisms, and/or the development of emphysema that manifest in human COPD. Many researchers now carry out relatively complex models, which mimic multiple chronic exposures. For example, Sussan et al. compared acute and chronic exposure to wood or cow dung PM in a murine model [25]. Acute exposure resulted in the production of pro-inflammatory cytokines, neutrophilic inflammation, and increased airway resistance and hyper-responsiveness, with PM from cow dung inducing greater responses than wood smoke PM. In the same study, subchronic exposures increased eosinophilic inflammation and destruction of alveoli tissue with wood smoke PM having greater activity. 

### 6.3. Controlled Human Exposure 

There have been many studies demonstrating a strong association between chronic exposure to biomass smoke and poor health outcomes [16,17,23,29]. However, it must be acknowledged that biomass smoke constitutes one of a number common and harmful household pollutants [51]. In vitro and animal studies directly assessing the impact of exposure to biomass smoke alone have already been discussed. However, there can be little doubt that human exposure models represent the most relevant and desirable model available to demonstrate a strong causal link between biomass smoke exposure and disease. For ethical reasons these can only be limited to relatively short term exposures and as such can only be used to examine acute effects of exposure. This, and current the lack of a standardized model, limit their usefulness. In addition, human exposure studies do not always yield consistent findings. Several recent studies examining any pro-inflammatory effects of biomass smoke exposure of otherwise healthy volunteers illustrate this. Two three-hour chamber exposures to incomplete combustion wood smoke (314 μg/m^3^) reduced inflammatory cells and mediators levels in broncho-alveolar lavage, whilst T-cells and mast cells were increased in the airway walls from endobronchial mucosal biopsies [68]; whereas Ghio and colleagues reported peripheral and lung neutrophilia in response to four fifteen-minute exposures to wood smoke (485 ± 84 μg/m) over a two-h period [69]. Disparate findings have also been observed when researchers assessed the effect of biomass smoke on peripheral inflammation, Burchiel et al. exposed people to hardwood smoke for 2 h (500 ug/m^3^), and then examined the response of peripheral blood mononuclear cells ex vivo [70]. They found that T cell proliferation and cytokine production in response to hardwood smoke exposure was highly variable from one individual to another, while the two responses in any individual were consistent. While after exposure to wood smoke (0.41 mg/m^3^) in a reconstructed Viking house for a one week stay [71], no adverse effects were demonstrable in participants with regard to measures of genotoxicity nor with inflammatory markers (serum C-reactive protein, IL6, IL8, TNF) nor with indicators of cardiovascular disease (cholesterol, triglycerides, and high-density lipoproteins levels). The number of circulating monocytes expressing CD31 were slightly increased, but not for the monocytes expressing CD11b, CD49d, and CD62L. 

It is probably unreasonable to expect consistency between the studies of acute human exposure given that relative amounts of active components will vary between sources and the inherent variability between individuals with regard to pulmonary and immune responses to noxious stimuli as a reflection of differences in general health status and life history of toxicant exposure.

## 7. Is Oxidative Damage the Major Mechanism of Biomass Smoke Induced COPD?

Oxidative stress can be conceived of as an imbalance between the burden of oxidizing species and the antioxidant defenses of cells and tissues. The imbalance being such that the cell and tissue defenses cannot deal with an increased burden of oxidants leaving an excess, which can target cell and tissue components leading to alterations or loss of their functions [72,73]. With biomass smoke exposure, this burden of oxidising species would be potentially derived from both components of the smoke [74] as well as the inflammatory cells recruited to the lungs [75]. However, caution must be taken when making any conclusions about the role of oxidative stress in the pathogenesis and/or pathology of any disease. For example, demonstration of increased oxidant production commonly relies upon reactions with indicator species such as dihydro-dichloro-fluorescein and dihydro-ethidium. The concern here is that in a competent cell host antioxidant defenses may successfully consume the same reactive oxidants in the absence of the competing reactions with the added indicator. Alternatively, researchers have shown either decreased levels of low molecular weight antioxidants or an increase in the levels of antioxidant enzymes in response to toxicant exposure. 

The changes do represent the result of (or response to) an increased ROS load but not necessarily an inadequate antioxidant response. The oxidative stress would only be supported by oxidative damage to host cell and tissue components given that there are a number of well-characterised and stable markers of such damage that researchers can measure. Ideally, studies would be designed to find a significant correlation between physiological parameters and either a depletion in antioxidant defence or markers of oxidative damage. However, association is not causation and such studies need to be supported by follow up work where interventions that specifically boost host antioxidant defence are matched with an amelioration or blunting of the effects of the toxicant on the functions assessed [65].

There have been several studies, which have assessed the association between oxidative stress and acute or chronic biomass exposure. Several studies have examined rural Indian women chronically exposed to biomass smoke. Dutta et al. demonstrated increased ROS production (as determined by increased oxidation of added dihydro-dichloro-fluorescein) but decreased superoxide dismutase (SOD) activity in epithelial and leukocytes isolated from sputum collected from such women [66]. The superoxide radical spontaneously dismutates to ROS hydrogen peroxide, while SOD catalyses the same reaction. Superoxide is a transition metal reductant which promotes the formation of Fenton-type ROS. Banerjee et al. [76] and Mukherjee and colleagues [77] found similar findings to those of Dutta et al. [78]. Further to this, Dutta et al. [79] compared leukocyte ROS production and erythrocyte SOD activity between Indian women who used liquid petroleum gas for cooking with those who cooked using biomass fuel. With regard to cells from women who were exposed to biomass smoke, ROS generation and SOD activity were, respectively, increased and decreased. Notably ROS generation was positively correlated, and SOD activity was inversely correlated with PM10 and PM2.5 levels in the women’s blood. Additionally, Mukherjee and colleagues also demonstrated evidence suggestive of oxidative attack upon DNA in the cells isolated from the sputum of rural Indian women exposed to biomass smoke [77]. The assay for oxidative attack upon DNA relies on an assay of DNA strand breakages. Also when comparing a group with biomass smoke-attributed COPD to matched healthy subjects, Ceylan et al. found a significant higher level of DNA strand in isolated leukocytes as well as increased serum levels of malondialdehyde and protein carbonyls (used as markers of lipid and protein oxidation, respectively) [70].

In contrast to these studies, in a group of Danish female and male university students exposed to biomass smoke in a reconstructed Viking-Age house for weekly periods Jensen et al. [80] found no significant evidence of increased DNA strand breakage in peripheral blood leukocytes. These latter results are not surprising noting that in healthy young men and women, short term exposure to wood smoke increased the level of the antioxidant glutathione in broncho-alveloar lavage fluid [81] highlighting the capacity of body to handle an increased oxidative burden through increasing its antioxidant defence. The contrast with the earlier cited studies [70,76] is that chronic exposure to biomass smoke may represent a persistent burden of ROS while antioxidant defence begins to fail resulting in cell and tissue damage.

Two recent students have examined associations between the measures of oxidative stress and lung function. Montano et al. [82] compared people with COPD attributed to biomass smoke exposure with healthy matched controls. In the biomass smoke-COPD group, all measures of lung function (FEV_1_, FVC and FEV_1_:FVC were significantly lower and there was a significant inverse correlation with regard to serum SOD activity [82]. However, this was not the case for other antioxidant enzymes (glutathione peroxidase, glutathione-S-transferase and glutathione reductase). In the same study, an inverse relationship was also found for serum malondialdehyde levels and lung function. The same inverse relationship between serum malondialdehyde levels (and serum SOD activity) and lung function were found in mothers and children exposed to biomass smoke [83].

Malondialdehyde is commonly used as a measure of oxidative stress specifically, as mentioned earlier, a measure of lipid peroxidation. However, there are several pathways by which malondialdehyde can be formed and there are available more specific, validated and stable markers of lipid peroxidation notably, F_2_-isoprostanes [84]. When F_2_-isoprostanes have been used as a marker of oxidative stress, there have been conflicting results. Increased urine excretion of 8-iso-prostaglandin_2α_ was demonstrated in nine healthy volunteers exposed to two 4-h periods of wood smoke with 1 week apart [85]. However, these findings contrast to the study of Commodore et al. in which the urinary levels of 8-iso-prostaglandin_2α_ and 8-hydroxy-2′-deoxyguanosine (a stable and direct marker of DNA oxidative damage) of Peruvian women who used wood fire stoves for cooking were assayed. While these researchers were able to demonstrate a weak positive correlation between cooking time and urinary 8-hydroxy-2′-deoxyguanosine levels, they found no significant differences in the urinary levels of these markers between a control group and an intervention group who used a modified stove designed to reduce biomass smoke exposure. Nor were these researchers able to demonstrate any significant positive correlation between the urine levels of these markers and measures of PM exposure [86].

It should be apparent that studies purported to examine oxidative stress in cases of biomass exposure need to be examined closely as to (i) the choice of assay; (ii) the characteristics of the biomass exposure; and (iii) the cells and tissues, which are being examined. A broader question is what would be the outcomes, in terms of better human health, if oxidative damage was identified as central to the adverse effects of biomass smoke? What would be the advantages of interventions involving boosting anti-oxidant defence over those working to reduce chronic domestic exposure to excessive amounts of biomass smoke?

## 8. Other Potential Mechanisms

McCarthy et al., found that in pneumocytes wood smoke activates the aryl hydrocarbon receptor [87]. They did not inhibit hydrocarbon receptor signaling so it is not possible to ascertain the extent of the involvement of signaling via this receptor in response to biomass smoke. Sussan et al. investigated signaling pathways of PM derived from wood and cow dung. They found that inflammation is primarily driven via Toll-Like Receptors (TLR) 4 and 2, and IL-1R, using a series of receptor knockout mice [25]. Since TLR 4 and 2 are both receptors for bacterial endotoxins, it is logical that biomass derived PM would activate these receptors. We interpret the signaling via IL-1R as a secondary signaling pathway since endotoxin activation of TLRs induces the production of IL-1 [88]. There are likely to be important differences between PM derived from biomass smoke (as used by Sussan et al.) and biomass smoke itself (as used by McCarthy et al.), which also contains gaseous components so it could in-fact be that these seemingly disparate findings are both correct, and different components activate different receptors and pathways. 

## 9. Biomass as a Risk Factor for COPD Exacerbations

Indoor pollutant exposures, including PM_2.5_ and NO_2_ have been associated with increased respiratory symptoms and risk of COPD exacerbations [89]. In addition, outdoor PM concentrations have been associated with an increase in COPD hospitalizations and mortality [90,91]. Outdoor nitrogen dioxide (NO_2_) exposure has also been linked to increased COPD morbidity, including higher rates of exacerbations [92,93]. In countries with low levels of biomass pollution, forest fires provide an opportunity to explore the effects of biomass on COPD exacerbations. Several studies have investigated the effects of forest fires on emergency department visits for COPD in New South Wales, Australia. Perhaps unsurprisingly these epidemiological studies all showed an increase in COPD exacerbations [94,95,96]. Using similar methodology, a smoke event was defined by an increase in the average citywide PM_10_ or PM_2.5_ to exceed the 99th percentile of the entire study period. Admissions for COPD were (OR 1.12, 95% CI 1.02, 1.24) [94] and (OR = 1.13, 95% CI = 1.05–1.22) [95]. A different analysis method was used by Morgan et al. which revealed that 10 microg/m increase in bushfire PM10 was associated with a 3.80% (1.40 to 6.26%) increase in COPD Admissions [96]. It is important to recognise that further studies are needed to fully understand how exposure to biomass smoke may precipitate COPD exacerbations globally, especially in situations which were constant with where exposure occurs.

## 10. How Should In Vitro and In Vivo Models of Biomass Smoke Induced COPD Be Carried Out?

Clear evidence of a causative link between exposure to biomass smoke and respiratory events and diseases is important as an impetus for programs that would directed to modifying the use of biomass fuels. Population studies can at best show strong associations between biomass exposure and adverse health outcomes but they cannot demonstrate causation. In vitro and in vivo studies allow the examination of the direct action of biomass smoke upon cell, tissue and organ function. However, this is not without its challenges, some of which have been already discussed. One of the greatest challenges to researchers investigating the effects of biomass smoke exposure using in vitro and in vivo models is the inherent variability of biomass fuels. In contrast, cigarette manufacturers attempt to provide a consistent product by adding chemical constituents such as flavorings and nicotine to cigarettes and researchers can elect to use research cigarettes such as 1R6F available from the Center for Tobacco Reference Products produced by the University of Kentucky. There are no reference biomass fuels available specifically made for research purposes. 

In-vitro biomass smoke exposure models are based on cigarette smoke exposure models, but the method of biomass smoke exposure varies from study to study. In Vitro, cells can be exposed to smoke directly, or to constituents of the smoke. In Vivo the airways are covered by a layer of liquid known as airway surface liquid. This thin layer of liquid regulates airway homeostasis by entrapping particulate matter, bacteria and other inhaled materials [97]. As such, many researchers reason that bioactive compounds in biomass smoke need to be soluble in order to pass this liquid layer and to act upon the underlying cells. Practically this can be achieved by bubbling the smoke through liquid to make biomass smoke extract (analogous to cigarette smoke extract). Variables in the generation of biomass smoke extract include the mass of biomass combusted, the temperature of combustion (e.g., if a furnace used), the source of the biomass, the rate of smoke passing through the medium, which medium is used, the amount of medium, the length of tubing from the biomass to the medium, the size of the vessel used to condition the medium with smoke, and the amount of time (if any) at the end of combustion which smoke is left in contact with the medium. 

In our experiments, we have taken a pragmatic approach. We use the same mass of biomass as the amount of tobacco found in 1 standard commercial cigarette (so that we can compare to cigarettes). We ignited the biomass using a gas lighter (to avoid chemicals found in matches), and allowed the biomass to burn unaided (representing an open fire pit), and drew the smoke at a rate which allowed combustion to occur over two minutes. The flow rate will control both the amount of oxygen supplied to the burning biomass and the exposure time if using a bubbling extraction system. We use a disposable collection system in which a 175 cm^2^ tissue culture flask which contains 25 mL of medium (without FBS to avoid bubbling), as residue from the smoke builds upon the flasks. The lack of protein in the medium, in particular albumin, is worth some discussion. It is known that direct exposure of albumin to cigarette smoke extract results in carbonlyation (a type of oxidative modification) [98]. It is not known if greater oxidative modifications of proteins would occur if they were contained in the medium at the time of harvest, or if the oxidatively modified proteins have different bioactivity compared to none modified proteins. Following 5 min of smoke absorption, the biomass extract solution is defined as 100% and immediately diluted to working concentrations (typically 1–10% in medium containing 0.1–10% FBS (depending upon the required growth conditions). This medium is then applied in vitro for up-to 72 h. One of the underappreciated aspects of in vitro treatment is that the “smoke” can leach from one well to another. This can result in control cell cultures having exposure and confounding results. This is by no means the ideal way to treat cells with biomass, but a methodology, which we have developed over a period of around 5 years. 

Earlier mention was made of the issues of working with cells submerged in culture medium. An alternative is to use systems where epithelial cells are cultured at an air liquid interface and expose these to gaseous biomass. McCarthy et al. made dung cigarettes, and utilised a commercial smoking machine (Baumgartner-Jaeger CSM2072i, CH Technologies, Westwood, NJ, USA) to generate smoke and expose small airway epithelial cells, which had been at air: liquid interface for 24 h [87]. The obvious drawback to this particular system is that cigarettes were made with a filter, as used in a tobacco cigarette. Exactly which components of dung biomass the filter depleted was not assessed. 

Other innovations researchers have used include: (i) using domestic wood stoves to generate biomass smoke, collecting the PM and adding that to the culture medium [68]; using biomass smoke derived from fuel burned in a barbecue grill, which is then infused into culture medium [59].

In addition to variations in the choice of fuel and how the smoke is generated, an additional variant in in vivo animal models of biomass smoke exposure is delivery of the smoke. Exposure can either be whole body [99] or nose only [100]. Vlahos and Bozinovski have recently reviewed the different delivery methods [101]. One aspect of exposure that may not be commonly considered is how the animals will be housed post-exposure. In whole body exposure regimens, components from the smoke will coat the fur of the animals, and this is can be later ingested via grooming behaviors [102]. In addition, some animals are coprophagic. Simple measures such as undertaking smoke exposure in a container that is different to where the animals are housed and also keeping smoke-exposed animals in separate cages from their unexposed littermates should be used.

## 11. Concluding Remarks

While smoking rates continue to decline, the burning of biomass for energy is not expected to do the same and may even increase because of it is a relatively cheap and, particularly in the developed world, more attractive to electricity and liquid petroleum gas. There is substantial evidence which links chronic exposure of excess amounts of biomass smoke to adverse health effects notably COPD. Providing a causative link through in vitro and in vivo studies remains a challenge largely due to technical problems, which include a lack of standardized sources of biomass smoke as well as no agreed protocol for its generation and delivery. The issues here can, in some cases, be contrasted as to what is available with regard to the same sorts of studies investigating cigarette smoke. There is also a marked paucity of data to understand the nature of exacerbations of biomass induced COPD. Given the projected rise in the mortality of COPD, the weight of evidence is still not available which would be the driving impetus for intervention programs that lead to modifications in the use of biomass fuel and also in controlling people’s exposure.

## Figures and Tables

**Table 1:** The most abundant constituents of wood smoke adapted from [39].

Pollutant	Physical State	Emissions for g/kg Wood
Carbon Monoxide	vapour	80–370
Methane	vapour	14–25
VOCs (C2–C7)	vapour	7–27
Substituted Furans	vapour	0.15–1.7
	vapour	
Benzene	vapour	0.6–4.0
	vapour	
*Alkyl Benzenes (including toluene)*	vapour	1–6
*Aldehydes (including Formaldehyde, Acrolein, Propionaldehyde, Butryaldehyde, Acetaldehyde, Furfural)*		0.6–5.4
Acetic Acid	vapour	1.8–2.4
Formic Acid	vapour	0.06–0.08
*Nitrogen Oxides (NO,NO_2_)*	vapour	0.2–0.9
Sulfur Dioxide	vapour	0.16–0.24
Methyl chloride	vapour	0.01–0.04
Napthalene	vapour	0.24–1.6
*Substituted Napthalenes*	vapour/particulate	0.3–2.1
Total Particle Mass	particulate	7–30
	particulate	
Particulate Organic Carbon	particulate	2–20
	particulate	
*Oxygenated PAHs*	vapour/particulate	0.15–1
*Oxygenated Monoaromatics (including Guaiacol (and derivatives), Phenol (and derivatives), Syringol (and derivatives), and Catechol (and derivatives)*		1–7
*PAHs (including Fluorene, Benzo(e)pyrene), Chrysene*	vapour particulate	<1 g
